# Inhibitory Effects of *Reynoutria japonica* Houtt. on Pain and Cartilage Breakdown in Osteoarthritis Based on Its Multifaceted Anti-Inflammatory Activity: An In Vivo and In Vitro Approach

**DOI:** 10.3390/ijms251910647

**Published:** 2024-10-03

**Authors:** Hee-Geun Jo, Chae Yun Baek, Juni Lee, Yeseul Hwang, Eunhye Baek, Aejin Song, Ho Sueb Song, Donghun Lee

**Affiliations:** 1 Department of Herbal Pharmacology, College of Korean Medicine, Gachon University, 1342 Seongnamdae-ro, Sujeong-gu, Seongnam-si 13120, Republic of Korea; jho3366@hanmail.net (H.-G.J.); cyning20@gachon.ac.kr (C.Y.B.);; 2 Naturalis Inc., 6 Daewangpangyo-ro, Bundang-gu, Seongnam-si 13549, Republic of Korea; 3 RexSoft Inc., 1 Gwanak-ro, Gwanak-gu, Seoul 08826, Republic of Korea; 4 Department of Acupuncture & Moxibustion Medicine, College of Korean Medicine, Gachon University, 1342 Seongnamdae-ro, Sujeong-gu, Seongnam-si 13306, Republic of Korea

**Keywords:** *Reynoutria japonica* Houtt., osteoarthritis, anti-inflammatory, chondroprotective, analgesic, East Asian herbal medicine

## Abstract

In the past 30 years, the number of years lived with disability due to osteoarthritis (OA) has doubled, making it an increasing global health burden. To address this issue, interventions that inhibit the progressive pathology driven by age-related low-grade inflammation, the primary mechanism of OA, are being actively pursued. Recent investigations have focused on modulating the age-related low-grade inflammatory pathology of this disease as a therapeutic target. However, no agent has successfully halted the disease’s progression or reversed its irreversible course. *Reynoutria japonica* Houtt. (RJ), a promising East Asian herbal medicine, has been utilized for several diseases due to its potent anti-inflammatory activity. This study aims to determine RJ’s capacity to inhibit OA symptoms and associated inflammation, exploring its potential for further development. In vivo and in vitro experiments demonstrated RJ’s anti-OA activity and modulation of multifaceted inflammatory targets. RJ significantly inhibited pain, gait deterioration, and cartilage destruction in a monosodium iodoacetate-induced OA rat model, with its analgesic effect further confirmed in an acetic acid-induced writhing model. RJ exhibited consistent anti-inflammatory activity against multiple targets in serum and cartilage of the OA rat model and lipopolysaccharide-induced RAW 264.7 cells. The inhibition of inflammatory cytokines, including interleukin-1β, interleukin-6, matrix metalloproteinase-13, tumor necrosis factor-α, and nitric oxide synthase 2, suggests that RJ’s alleviation of OA manifestations relates to its multifaceted anti-inflammatory activity. These results indicate that RJ merits further investigation as a disease-modifying drug candidate targeting OA’s inflammatory pathology. To further characterize the pharmacological properties of RJ, future studies with expanded designs are warranted.

## 1. Introduction

Osteoarthritis (OA) is a leading cause of knee pain, affecting over 6 million people worldwide with chronic joint discomfort, impaired physical function, and limited mobility [1]. OA causes not only the progressive destruction of articular cartilage and subchondral bone, but also the irreversible breakdown of the entire joint structure, including the synovial membrane and surrounding soft tissues [2,3]. With an aging and increasingly obese global population, OA is becoming more prevalent, incurring substantial direct healthcare and societal costs [4,5]. The etiology of these pathologies is considered to be multifactorial, with several factors proposed as potential contributors, including work intensity, recurrent knee injuries from sports, and other musculoskeletal injuries. However, further research is required to determine whether these factors have a direct causal relationship with OA pathology [6,7,8]. Recent reports highlighting significant disparities in OA burden between high-income and low-income countries underscore the urgency of addressing this issue [9]. Considering the rising incidence of OA, effective treatments that can halt or reverse disease progression remain elusive, emphasizing the urgent need for innovative therapeutic interventions. The pathogenesis and pathology of OA remain incompletely understood. Recent research indicates that OA may be driven by inflammaging—a process that combines low-grade systemic inflammation with aging—in addition to mechanical wear and tear [10,11]. Current understanding posits inflammaging, characterized by increased secretion of senescence-associated secretory phenotype (SASP) factors due to cellular senescence, to be a key pathogenic mechanism in OA [12,13]. Concurrently, other research indicates that mechanical joint overload or underload interacts extensively with local and systemic pro- and anti-inflammatory mediators [14]. These factors collectively expose joint chondrocytes and synovial fibroblasts to inflammatory mediators such as interleukin (IL)-1β, IL-6, and tumor necrosis factor (TNF)-α, which subsequently induce the production of additional articular tissue-degrading enzymes and pro-inflammatory factors, including matrix metalloproteinases (MMPs), potentially initiating OA pathogenesis [15,16]. Therapeutic approaches targeting inflammatory pathways have garnered interest as potential disease-modifying treatments, given the significant role of chronic inflammation in osteoarthritis. For example, previous studies have reported that IL-1 receptor antagonists exert immunosuppressive effects on mesenchymal stem cells, inducing M2 macrophage polarization and reducing the antigen-presenting properties of dendritic cells. This, in turn, inhibits inflammatory activation and alleviates joint disease [17]. Consequently, recent OA therapeutic developments have focused on identifying candidate drugs capable of modulating the multifaceted, low-grade inflammatory state affecting the entire body.

Although a variety of therapeutic modalities are currently available for the symptomatic relief of osteoarthritis (OA) pain, effective treatments that demonstrate the ability to stop or reverse the ongoing pathological deterioration associated with the disease remain in the research phase. For that reason, most international guidelines recommend non-pharmacological approaches, such as therapeutic exercise, lifestyle modification, and weight loss, as first-line interventions [18]. Intra-articular injections of glucocorticoids, viscosupplementation, platelet-rich plasma, and mesenchymal stem cells have been extensively investigated as alternative therapies; however, recent clinical studies have questioned their long-term efficacy [19,20,21]. Notably, glucocorticoids, despite their potent anti-inflammatory activity, have failed to demonstrate superiority over physical therapy in OA treatment in clinical trials and are associated with risks of severe adverse events, including steroid-induced osteonecrosis [22,23]. Nonsteroidal anti-inflammatory drugs (NSAIDs) are widely utilized as a substitute therapy, but their extensive gastrointestinal, cardiovascular, and renal toxicity cannot be disregarded in terms of long-term safety, given OA’s chronic dosing requirements [24,25,26]. Consequently, developing disease-modifying OA drugs (DMOADs) that can ameliorate OA symptoms and underlying pathologies is a major research priority [27,28,29,30]. However, clinical trials and regulatory approvals for this new drug class have not yet succeeded, and the repurposing of existing drugs, despite initial promise, has not yielded positive outcomes.

OA therapeutic development necessitates the modulation of multiple inflammation-related targets [12,13]. Natural products are considered to be promising OA therapeutic candidates due to their unique multicomponent pharmacology and multifaceted anti-inflammatory inhibition [31,32]. Among global natural product sources, East Asian herbal medicine (EAHM) offers comparative advantages for drug discovery, with hundreds of medicinal herbs listed in major East Asian pharmacopeias and millennia of documented use [32,33]. The last decade has witnessed a surge in research on EAHM effects and mechanisms, particularly regarding their correlation with OA and rheumatoid arthritis inflammatory pathology [33,34,35,36,37,38,39,40,41,42]. *Reynoutria japonica* Houtt. (RJ) emerges as a promising medicinal plant with diverse pharmacological activities, including potent anti-inflammatory, antioxidant, antiviral, and neuroprotective effects, reportedly treating cerebral ischemia, inflammatory bowel disease, asthma, myocardial hypertrophy, and joint diseases [43]. RJ contains stilbene compounds, including resveratrol and polydatin, and anthraquinone compounds, such as emodin, as primary active ingredients. Resveratrol and emodin have demonstrated promising effects on OA in several nonclinical studies [44,45,46]. However, a recent clinical trial showed that resveratrol was not superior to placebo in controlling OA pain [47]. These findings suggest that natural product research for DMOAD candidates should focus on whole plant extract multi-component, multi-target effects rather than single compounds. Despite RJ’s potent anti-inflammatory effects and pharmacological activity in multiple diseases, in-depth studies on its impact in OA remain insufficient.

In drawing on the insights gleaned from these preceding studies, we postulate that RJ has the potential to serve as a DMOAD candidate capable of partially suppressing pain and cartilage destruction through its capacity to inhibit the inflammatory pathology of multifactorial OA. This study aimed to screen RJ as a DMOAD candidate to determine its worthiness for further investigation. We report the results below, including its multi-target anti-inflammatory activity in an in vitro model and its efficacy in suppressing OA symptoms, such as pain and cartilage erosion, in an in vivo OA model.

## 2. Results

### 2.1. HPLC Chromatogram Analysis

**Figure 1:**
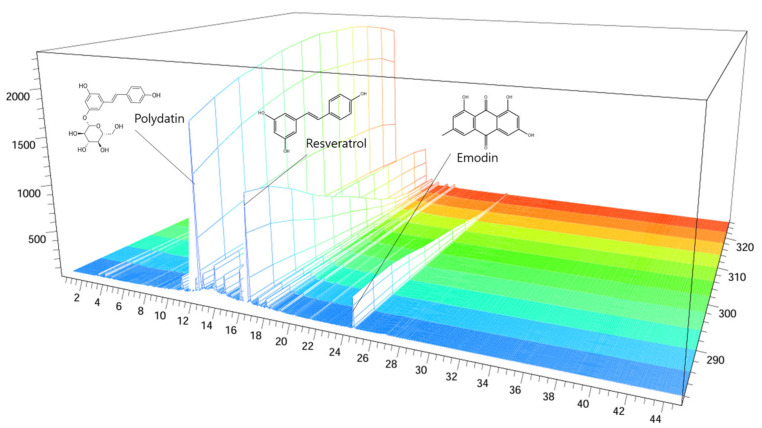
HPLC chromatogram of the RJ extract at 280 nm: polydatin, resveratrol, and emodin retention time = 11.997 min, 15.437 min, and 24.166 min, respectively. The x-axis shows the retention time; the y-axis shows the wavelength; the z-axis shows the absorbance unit. HPLC: High-performance liquid chromatography; RJ: *Reynoutria japonica* Houtt.

The polydatin, resveratrol, and emodin in *Reynoutria japonica* (RJ) were 45.942, 4.977, and 95.848 mg/g, respectively. Figure 1 indicates the UV spectra (280 nm) of polydatin, resveratrol, and emodin. The sample used in the following experiments were used in all subsequent studies.

### 2.2. Analgesic Effects on the RJ with Weight-Bearing Capacity

**Figure 2:**
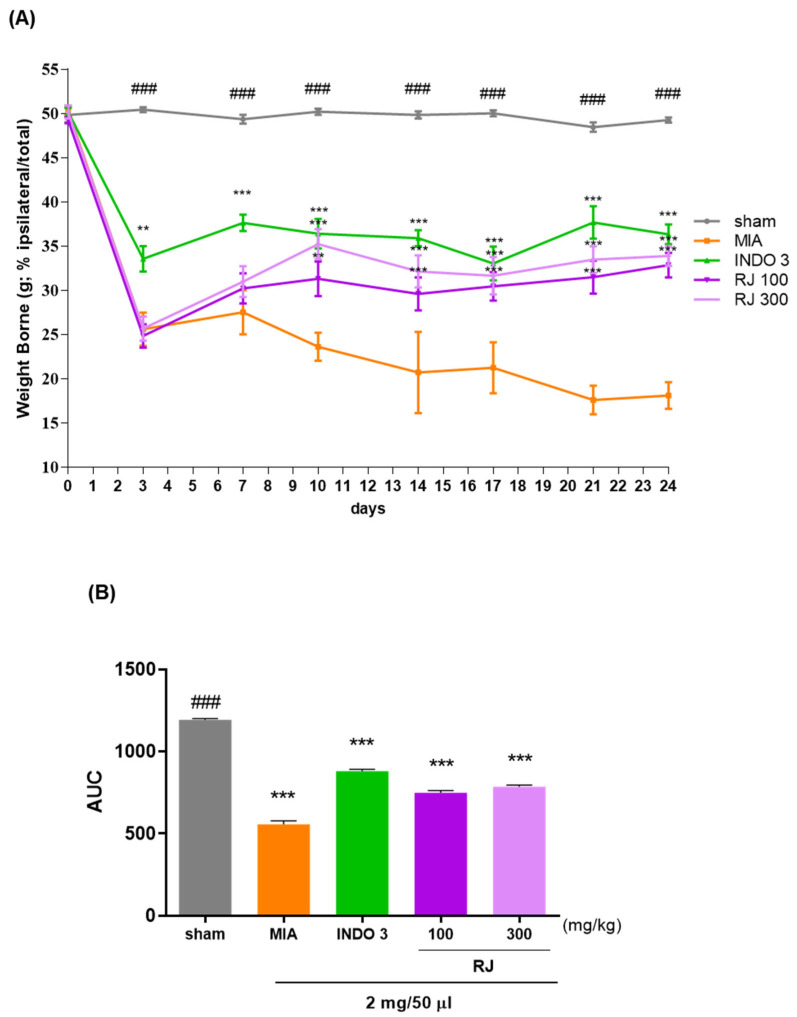
Assessment the discomfort about the weight-bearing capacity of the OA-induced model; (**A**) the weight bearing of OA rats in the RJ 100, RJ 300, and INDO 3 groups (n = 9) for 0 to 24 days; (**B**) the AUC was recorded using the incapacitance meter. ** *p* < 0.01 vs. MIA, *** *p* < 0.001 vs. MIA, ### *p* < 0.001 vs. sham Tukey’s multiple comparison test after Two-way ANOVA. AUC: area under the curve; INDO 3: indomethacin 3 mg/kg; MIA: monosodium iodoacetate, RJ: *Reynoutria japonica* Houtt., sham: non-treated group.

The weight-bearing capacity of the hind legs was recorded using a pressureless instrument to assess the discomfort and analgesic effect of putting weight on the hind limb, which allowed us to determine if the pain caused by OA improved after treatment. The weight-bearing capacity of the right and left legs was measured over a 24-day period. Samples from each group were administered daily. Weight-bearing ratios were significantly reduced after 10 days in the monosodium iodoacetate (MIA) group (23.64), followed by similar effects in the indomethacin 3 mg/kg (INDO 3) group (36.44), especially in the RJ 300 group (35.25) (Figure 2A,B). This suggests that RJ treatment improves gait in OA-induced rats.

### 2.3. Cartilage Degradation in OA-Induced Model

**Figure 3:**
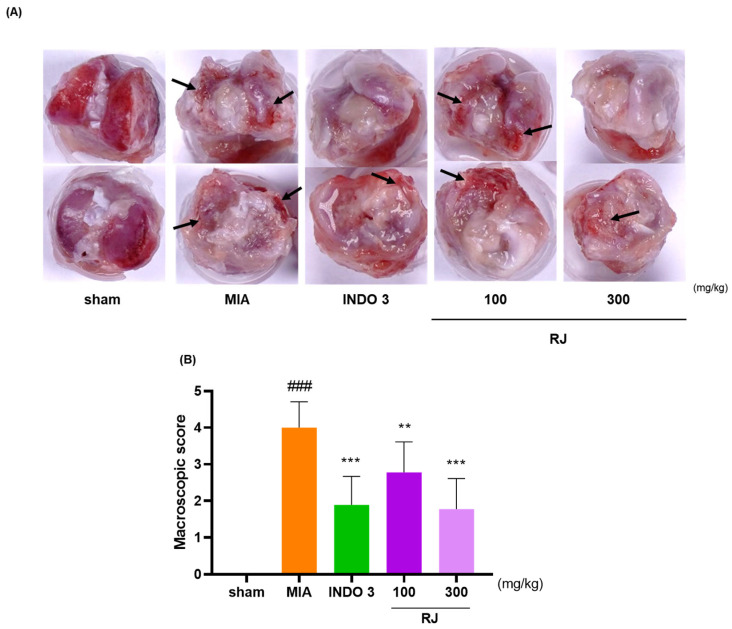
Photos of the right knee cartilage of OA-induced model. INDO 3, RJ 100, and RJ 300 (*n* = 9) were administered to OA-induced rats. (**A**) Representative images showing cartilage degradation. Arrows mean the cartilage-damaged site. (**B**) Macroscopic score. Grade 0: Typical appearance of the cartilage surface; grade 1: Slight yellow discoloration or mild fibrillation; grade 2: Erosion in the middle or superficial layer of cartilage is visible; grade 3: Severe deterioration reaching the subchondral bone; grade 4: Massive damage and extensive disclosure of subchondral bone. ** *p* < 0.01 vs. MIA, *** *p* < 0.001 vs. MIA using 1-way ANOVA and Dunnett’s test, ### *p* < 0.001 vs. sham by Unpaired *T*-test by Two-tailed *p* value. INDO 3: indomethacin 3 mg/kg; MIA: monosodium iodoacetate, RJ: *Reynoutria japonica* Houtt., sham: non-treated group.

After 24 days, the right knee cartilage of the OA-induced model was harvested and photographed. It was observed that RJ could effectively inhibit the cartilage degradation using the MIA solution. The cartilage in the sham group appeared glossier compared with the MIA group (Figure 3A). The joint surface in the MIA group lost polish, became rough, and exhibited erosion in certain areas. Based on macroscopic scoring, the degree of cartilage degeneration was significantly improved in rats treated with RJ and INDO 3 (Figure 3B). Notably, both RJ and INDO 3 treatments similarly repaired the damaged cartilage areas. The results suggest that RJ treatment can prevent sustained damage to knee cartilage in OA-induced rats.

### 2.4. Gait Analysis of OA-Induced Model

**Figure 4:**
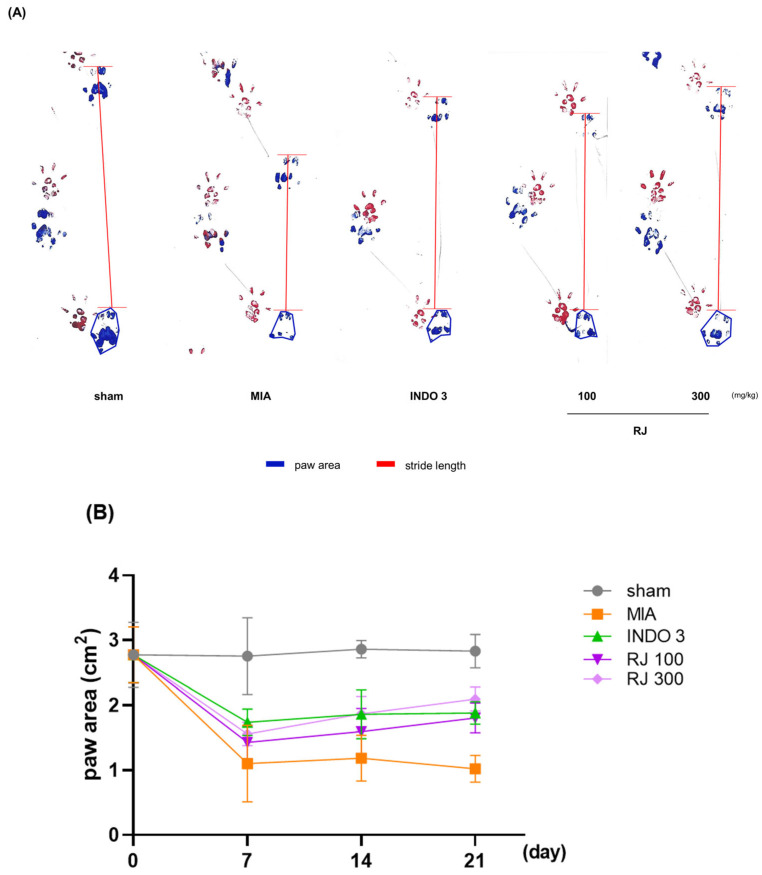

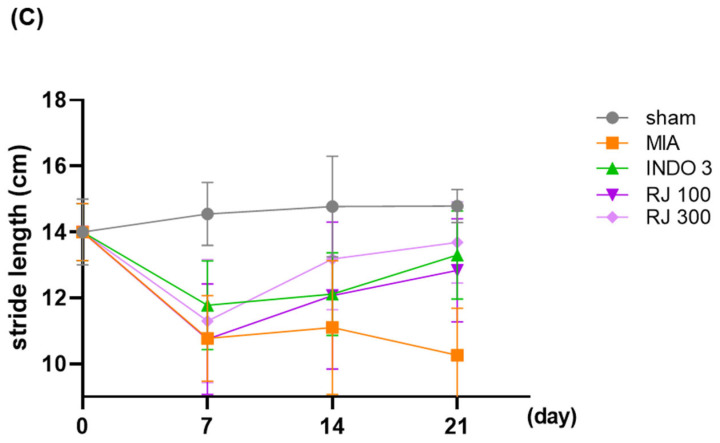
Gait analysis of OA-induced model during 21 days. (**A**) Representative photo indicated to footprints. (**B**) Paw area (blue line) and (**C**) stride length (red line) was analyzed on day 0, 7, 14, and 21 in OA-induced rats (n = 9). INDO 3: indomethacin 3 mg/kg; MIA: monosodium iodoacetate, RJ: *Reynoutria japonica* Houtt., sham: non-treated group.

To assess the pain of the OA-induced rats, their walking ability was checked. Representative footprints from the gait analysis are shown in Figure 4. Dynamic gait analyses were performed on days 7, 14, and 21 of 24 days of OA induction and were analyzed by measuring paw area and stride length. Paw area and stride length were significantly different between the MIA group and the INDO 3, RJ 100, and RJ 300 groups on day 21, and RJ 100 and RJ 300 had similar effects to INDO (Figure 4B,C). The results suggest that RJ treatment improves mobility and suppresses pain in OA-induced rats.

### 2.5. Inflammatory Cytokine Levels Analysis in OA-Induced Rats

**Figure 5:**
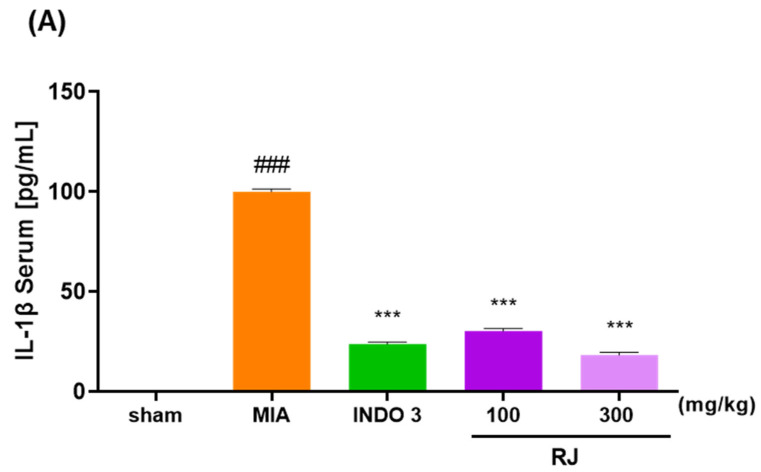

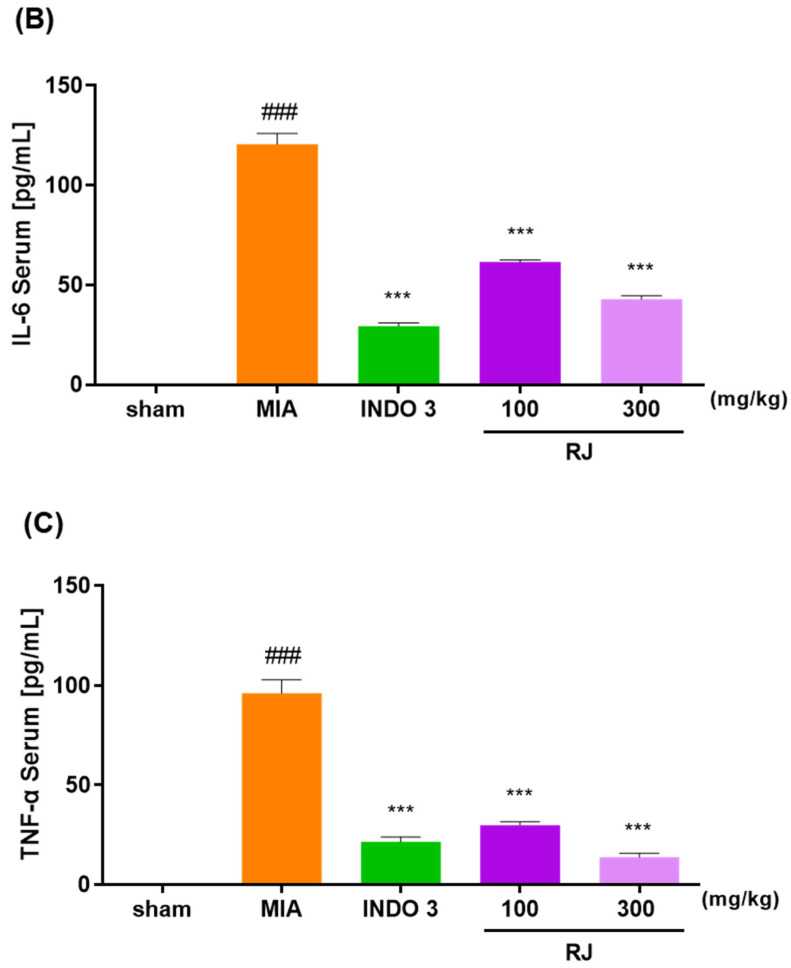
Effect of cytokines level in the RJ groups. (**A**) IL-1β, (**B**) IL-6, and (**C**) TNF-α expression level in OA-induced rats. OA models were treated RJ 100 and RJ 300 (n = 9) during 0–24 days. *** *p* < 0.001 vs. MIA by One-way ANOVA and Dunnett’s test, ### *p* < 0.001 vs. sham by Unpaired *T*-test by Two-tailed *p* value. INDO 3: indomethacin 3 mg/kg; MIA: monosodium iodoacetate, RJ: *Reynoutria japonica* Houtt., sham: non-treated group.

The RAW264.7 cells are a commonly used to inflammatory responses, and in this study, we assessed the toxicity of the RJ on the cells and inhibited NO production in LPS-treated cells to determine if the RJ had anti-inflammatory effects. The RJ group had significantly reduced serum levels of IL-1β, IL-6, and TNF-α compared to the MIA group, and these reductions were dose-dependent. In particular, RJ 300 reduced IL-1β (18.36) and TNF-α (13.84) levels to significantly lower levels than those observed in the INDO 3 group (23.86 and 21.52) (Figure 5).

### 2.6. Analgesic Effects in the AIW Models

**Figure 6:**
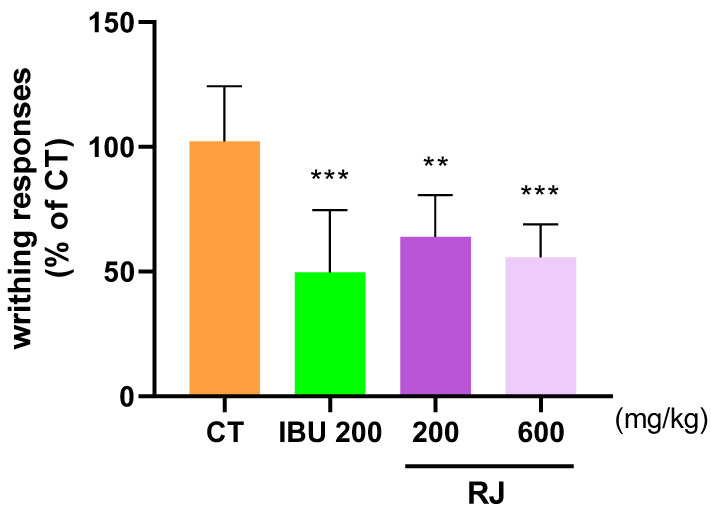
Analgesic effect of RJ in AIW models. Mice were treated with RJ 200, RJ 600, and IBU 200 (n = 8). All mice were injected with 0.7% acetic acid intraperitoneally 10 min prior to the recorded. ** *p* < 0.01 vs. CT, *** *p* < 0.001 vs. CT by One-way ANOVA and Dunnett’s test. CT: control, IBU 200: ibuprofen 200 mg/kg; RJ: *Reynoutria japonica* Houtt.

The analgesic effect of RJ was evaluated using the AIW response to assess the level of pain relief. Specifically, the writhing behavior of mice injected with acetic acid was recorded after 10 min, and the CT group showed an average of 100 writhing behavior. The IBU 200 and RJ 600 groups showed mean values of 49.65 and 55.66, respectively, indicating that the analgesic effect of RJ 600 was similar to that of IBU 200 (Figure 6). IBU was used as a positive control.

### 2.7. Cell Viability and NO Levels Analysis

**Figure 7:**
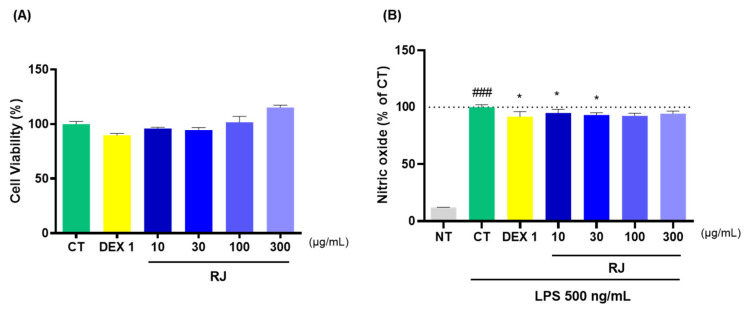
Effects of RJ using RAW264.7 cells. (**A**) Cell viability (%) and (**B**) NO production. * *p* < 0.05 vs. CT by 1-way ANOVA and Dunnett’s test, ### *p* < 0.001 vs. NT by Unpaired *T*-test by Two-tailed *p* value. CT: control, DEX 1: dexamethasone 1 μg/mL, LPS: lipopolysaccharide, NT: non-treated, RJ: *Reynoutria japonica* Houtt.

To assess RJ toxicity, RAW264.7 cells were treated with various doses of RJ, and cytotoxicity was evaluated using the EzCytox. RJ showed no signs of cytotoxicity (Figure 7A). To examine the anti-inflammatory effects of RJ, RAW264.7 cells were incubated with LPS to induce NO production. Especially, RJ reduced LPS-induced NO production, although no reduction in NO expression levels was measured in the RJ 300 group compared to the CT group (Figure 7B).

### 2.8. Effects on Anti Inflammatory in the RJ

**Figure 8:**
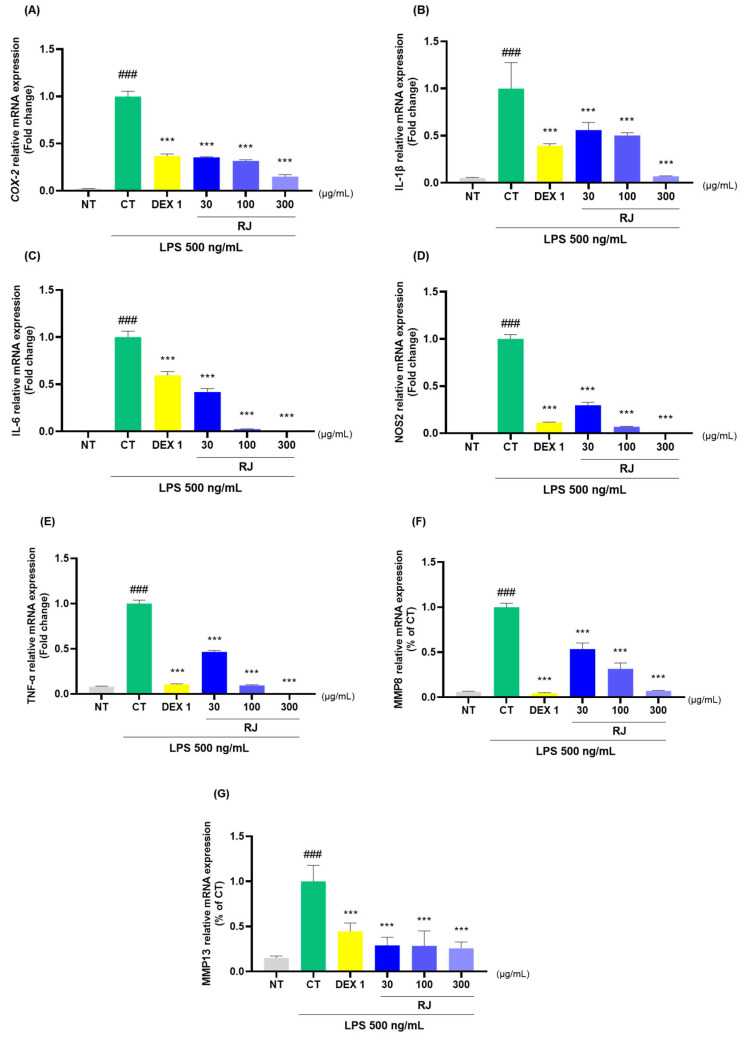

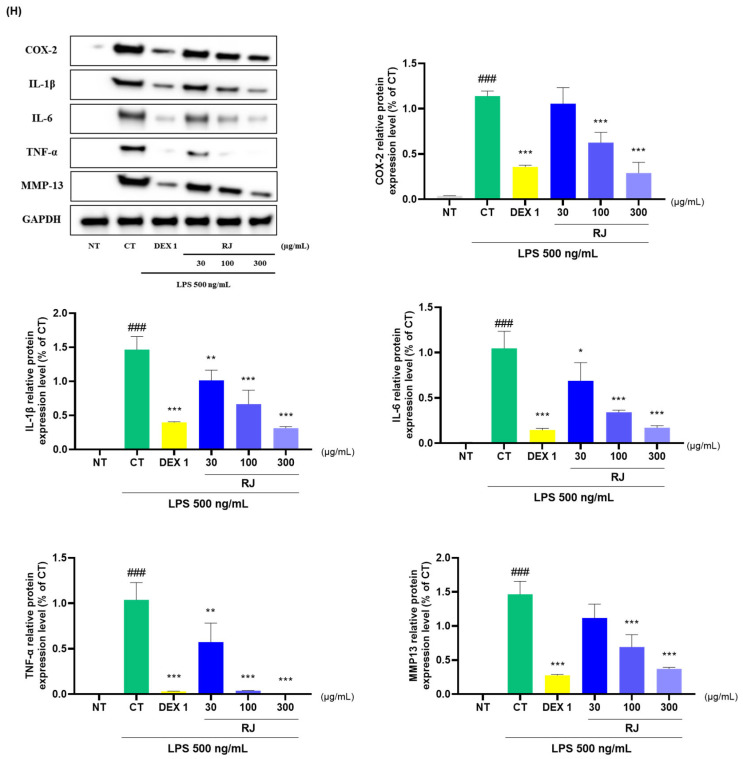
Analysis of cytokine expression levels in RAW264.7. (**A**–**G**) mRNA analysis of COX-2, IL-1β, IL-6, NOS2, TNF-α, MMP8, and MMP13; (**H**) protein analysis of COX-2, IL-1β, IL-6, TNF-α, and MMP13. The cells were incubated with LPS and DEX 1, RJ 30, RJ 100, and RJ 300 during 24 h. * *p* < 0.05 vs. CT by 1-way ANOVA and Dunnett’s test, ** *p* < 0.01 vs. CT, *** *p* < 0.001 vs. CT, ### *p* < 0.001 vs. NT by Unpaired *T*-test by Two-tailed *p* value. CT: control, DEX 1: dexamethasone 1 μg/mL, LPS: lipopolysaccharide, NT: non-treated, RJ: *Reynoutria japonica* Houtt.

RJ and DEX 1 groups significantly reduced the mRNA levels of COX-2, IL-1β, IL-6, NOS2, TNF-α, MMP8, and MMP13 (Figure 8A–G). Additionally, RJ treatment in LPS-stimulated RAW264.7 cells led to a decrease in the protein expression levels of these cytokines (Figure 8H). Western blot analysis further confirmed that RJ decreased the expression of COX-2, IL-1β, IL-6, TNF-α, and MMP13. Notably, RJ indicated anti-inflammatory effects comparable with DEX 1 for all cytokines.

### 2.9. Cytokine Levels Analysis in OA-Induced Model

**Figure 9:**
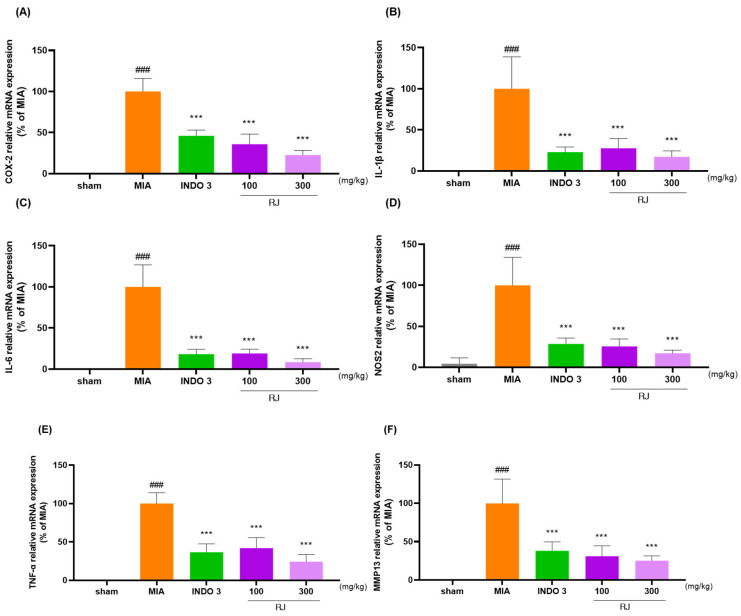

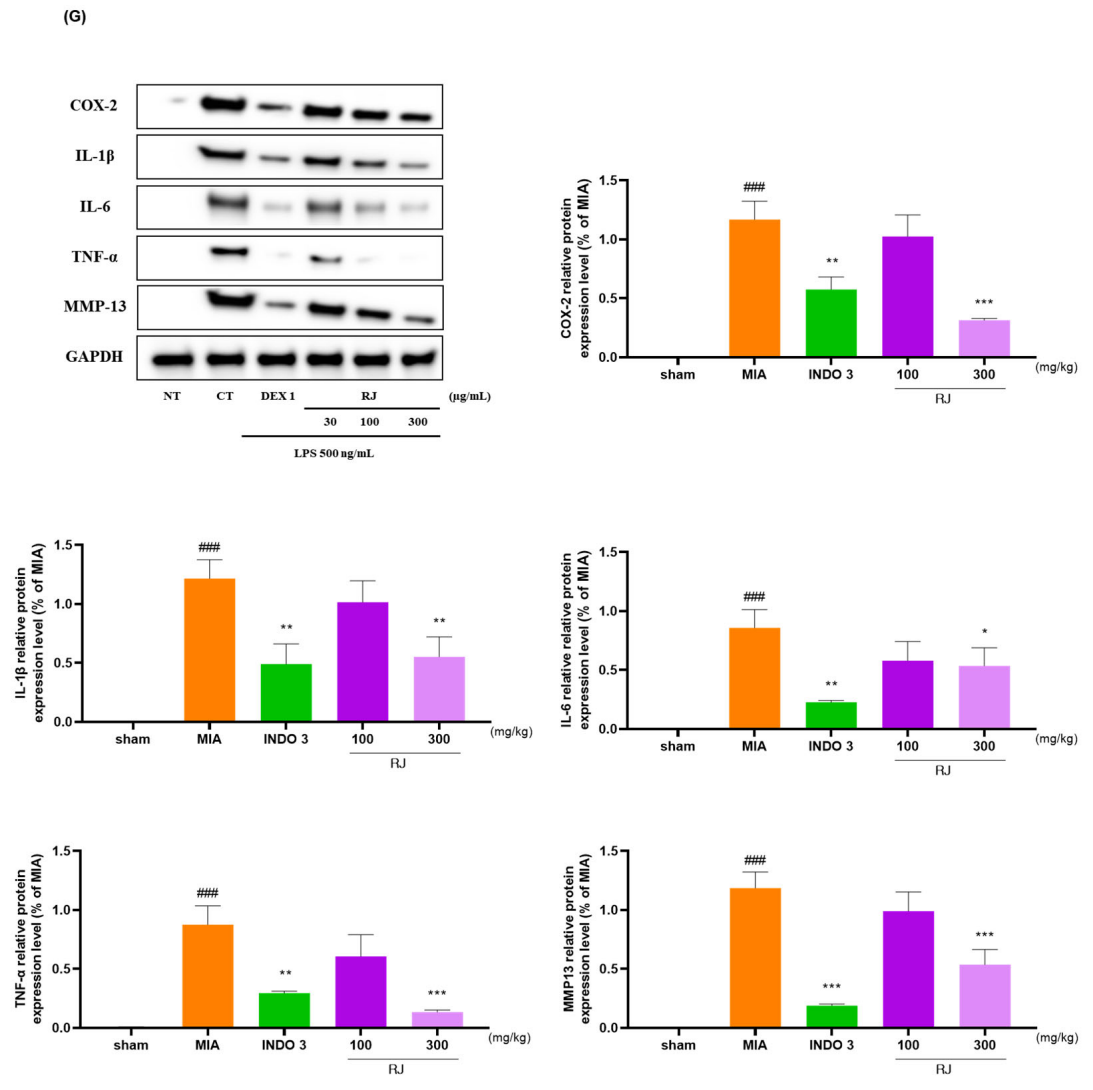
RJ groups reduced inflammatory cytokine levels in OA-induced models. (**A**–**F**) mRNA levels of COX-2, IL-1β, IL-6, NOS2, TNF-α, and MMP13 analyzed using qRT-PCR; (**G**) protein expression levels of COX-2, IL-1β, IL-6, TNF-α, and MMP13 analyzed using Western blot analysis. ### *p* < 0.001 vs. sham by Unpaired *T*-test by Two-tailed *p* value, * *p* < 0.05 vs. MIA, ** *p* < 0.01 vs. MIA, *** *p* < 0.001 vs. MIA using one-way ANOVA and Dunnett’s test. INDO 3: indomethacin 3 mg/kg; MIA: monosodium iodoacetate, RJ: *Reynoutria japonica* Houtt., sham: non-treated group.

RJ significantly reduced the mRNA and protein levels of COX-2, IL-1β, IL-6, NOS2, TNF-α, and MMP13 in OA-induced rat’s cartilage compared to the expression levels in OA-induced models (Figure 9A–F). The downgrade effects of RJ on COX-2, IL-1β, IL-6, TNF-α, and MMP13 in OA rats were indicated through Western blot analysis (Figure 9G).

## 3. Discussion

As shown in the results, RJ significantly inhibited pain, associated dysfunction, and cartilage destruction in animal models of OA. It also demonstrated significant anti-inflammatory effects on multiple inflammation-related targets in cartilage in vivo and in a RAW 264.7 cell-based in vitro model. These effects were consistent across multiple experimental designs, dose-dependent, and significantly superior to active controls such as NSAIDs. Furthermore, research has demonstrated that RJ exhibits superior efficacy compared to glucocorticoids for certain cytokines, including COX-2, IL-1β, and TNF-α. These results suggest the promise of RJ as a DMOAD based on its multi-targeted activity against multiple OA-related inflammatory pathologies. While previous studies were limited to predicting the potential anti-OA effects of RJ or evaluating relevant activities based on a single compound, this study specifically confirmed the multi-component anti-OA activity of RJ as a whole extract.

In this study, polydatin (PubChem CID 5281718), emodin (PubChem CID 3220), and resveratrol (PubChem CID 445154), widely recognized as the main components of RJ, were identified by HPLC-UV analysis. Polydatin, a natural polyphenol with a chemical structure similar to Resveratrol, is noted for its ability to exert multitargeted effects on various organ systems, including the cardiovascular, central nervous, and musculoskeletal systems, despite being a single compound [48]. Particularly relevant to this study, polydatin has demonstrated dose-dependent anti-inflammatory activity in an in vitro model targeting TNF-α, IL-1β/8, cyclooxygenase-2 (COX-2), and prostaglandin E2 (PGE2). Anti-inflammatory and chondroprotective effects mediated by the nuclear factor-kappa B (NF-κB) and Nrf2/haem oxygenase-1 (HO-1) pathways have also been reported in a mouse model of OA with surgically induced medial meniscus instability [49,50]. These findings from previous studies align with our observations, as they are consistent with the effects of RJ in the present study. Emodin, a natural anthraquinone, has been extensively studied for its anti-inflammatory, anti-apoptotic, and antioxidant activities [51]. Like polydatin, emodin has shown therapeutic potential across various diseases, including osteoporosis, diabetes, immune disorders, kidney disease, and neurological disorders [52]. Notably, emodin has also exhibited potent synergistic anti-inflammatory properties when combined with other bioactive compounds, potentially expanding its applications in chronic inflammatory diseases [53,54]. These anti-inflammatory activities directly support the recently reported chondroprotective effects of emodin, which involve targeting cartilage matrix degradation and mitigating subchondral bone microstructure destruction in osteoporotic OA models [45,55].

The bioactivity of the major RJ constituents reported in previous studies is consistent with the observations in this study. However, as noted in the Introduction regarding the failed clinical trial of Resveratrol for OA, it is challenging to extrapolate the preclinical effects and mechanisms of individual components to humans. Furthermore, the potential indications of most medicinal herbs are based on aggregated information from human studies using whole extracts. In light of this, the present study explores the potential of RJ crude extracts as candidate materials for DMOADs from a holistic perspective based on their effects. Recent studies have demonstrated that multicomponent combinations of natural products can mediate pharmacological synergistic effects that are difficult to achieve with single components alone, strongly suggesting that future research should not be limited to identifying the mechanisms of individual components [56]. Regarding multicomponent synergy, an important study investigated the effects of RJ and key individual components in a mouse model of ulcerative colitis. The study found that polydatin, resveratrol, and emodin were less effective at inhibiting cytokine activity and NF-κB signaling when used individually, compared to the combination of these three components or RJ whole extract [57]. Considering these factors, although the broad anti-inflammatory and anti-OA effects of the major RJ components are well supported by our findings, we believe that future research should focus on elucidating the multitargeted and multicomponent effects of all RJ constituents on OA pathology.

In the animal experimental part of this study, the MIA-induced OA rat model was employed to evaluate the OA-specific pain suppression, functional improvement, and chondroprotective activity of RJ. The analgesic effect was separately assessed using the acetic acid-induced writhing model for cross-validation [58]. The MIA model is currently regarded as a standard model that best simulates the progressive OA observed in human clinical findings, with chondrocyte death, neovascularization, subchondral bone necrosis, and collapse induced by joint injection of MIA accurately reflecting the pathophysiology of OA [59,60]. In this model, RJ significantly improved gait impairment and reduced pain in both the 100 mg/kg and 300 mg/kg groups, with the 300 mg/kg group showing superior functional improvement over time compared to the active control, indomethacin. Additionally, RJ significantly attenuated cartilage destruction in OA rats at all doses, with the 300 mg/kg group demonstrating better effects than the active control. The analgesic activity of RJ was further corroborated by the writhing model. Beyond these symptomatic effects, RJ significantly inhibited serum levels of IL-1β, IL-6, and TNF-α in MIA rats, as well as the mRNA expression of IL-1β, IL-6, TNF-α, NOS2, and MMP13 in the joint cartilage of these animals. RJ’s potent anti-inflammatory activity targeting IL-1β, IL-6, and TNF-α, and its efficacy in animal models of inflammatory diseases, are well documented [43]. Moreover, NOS2 is a key mediator of chondrolysis and cell damage in inflammation-based OA, and its inhibition has been shown to confer chondroprotective effects [61]. MMP13, which is upregulated in the joint by IL-1 and TNF, leading to articular cartilage destruction and synovitis, is widely studied as a promising target for several DMOAD candidates due to its OA-specific properties [62]. Taken together, the in vivo results of this study and previous research provide substantial evidence to infer that RJ has the potential to inhibit the multifaceted manifestations of OA through its anti-inflammatory activity targeting key pathways.

In the in vitro part of the study, we cross-validated the multifaceted anti-inflammatory activity of RJ by assessing its effects on mRNA and protein expression in LPS-treated RAW264.7 cells. The results consistently supported the findings from the serum and cartilage tissue of the in vivo model rats, showing inhibition of IL-1β, IL-6, TNF-α, NOS2, and MMP13, with efficacy comparable to the potent anti-inflammatory control, dexamethasone. Notably, significant anti-inflammatory activity against COX2 and MMP8 was observed, which was not confirmed in vivo. Inhibition of COX2 reduces the production of prostaglandins, thereby controlling inflammation and pain, and selective COX2 inhibitors are perceived to have a better safety profile than non-selective NSAIDs [26]. However, over the last two decades, COX2 inhibitors have been reported to increase the risk of myocardial infarction and death in a dose-dependent manner, with this risk escalating within just a few weeks [63]. In light of these findings, further studies on the safety profile of RJ and in-depth research on its COX2 inhibitory activity may be warranted to explore its potential as a safer analgesic drug compared to selective COX2 inhibitors. Additionally, previous studies showing that diacerein, a widely used IL-1-targeted OA symptomatic drug, alleviates periodontitis-induced gingival mucosa inflammation in rats and reduces MMP8 expression via IL-1β inhibition may partially explain the correlation between RJ’s MMP8 inhibition and OA symptomatic relief observed in this study [64]. Other studies on the bioactivity of natural flavonoids have also reported that luteolin exerts anti-OA effects by inhibiting IL-1β-induced cartilage inflammation and subsequently reducing MMP8 production [65]. Overall, RJ demonstrated superior anti-inflammatory effects compared to active controls against key pro-inflammatory mediators both in vitro and in vivo, with many of these targets being directly involved in the pain and cartilage destruction characteristic of OA. These results suggest that RJ is a promising drug candidate for modulating the pathology of OA through its multitargeted activity against systemic low-grade inflammation induced by aging, warranting further investigation.

This study screened RJ to determine its merit for further development as a drug candidate capable of fundamentally modulating OA’s chronic progressive pathology. Despite the positive results, future studies must address the following limitations to validate the above hypotheses. Firstly, the anti-OA effects of RJ as a whole extract remain unstudied. While some active components have shown promising activity, recent negative clinical trial results for Resveratrol indicate that RJ’s therapeutic potential cannot be confirmed solely by these findings. Studies with expanded designs reflecting a broader range of targets and signaling pathways are necessary to elucidate RJ whole extract’s OA pathology modulation mechanisms. Secondly, the synergistic effects of major compounds that contribute to RJ’s efficacy as a total, rather than as individual components, require further characterization. Achieving this necessitates bioinformatics approaches to predict pharmacokinetic properties and binding affinities of each compound to essential targets, in addition to expanded experimental studies. Thirdly, this study did not determine the optimal dose for RJ’s anti-inflammatory and anti-OA effects, making this a priority for future research. Finally, given the chronic nature of OA, which requires long-term treatment, the safety profile of RJ is critical. Verifying whether RJ can overcome existing drugs’ safety limitations through appropriate toxicity studies is essential before advancing to human clinical trials. Notably, the optimal dose of RJ was not determined in this study, limiting the clinical applicability of the findings. Additionally, determining the ideal dosing period in future studies is crucial to ensuring efficacy while minimizing potential toxicity.

## 4. Materials and Methods

All experiments in this study were conducted in accordance with the ARRIVE 2.0 guidelines.

### 4.1. Sample Preparation

The raw material used in the experiments was a 30% ethanol reflux extraction of *Reynoutria japonica* Houtt. (RJ) roots acquired from Yaksudang Pharmaceutical Co., Ltd. (Seoul, Republic of Korea). Professor Donghun Lee of Gachon University’s Department of Herbal Medicine, College of Korean Medicine, deposited the voucher specimen (No. D200915011).

### 4.2. Animal

The animals used in the experiments were provided by DBL, Inc. (Seoul, Republic of Korea) as male Sprague–Dawley (SD) rats (190–210 g) about the OA model and male ICR mice (30–40 g) for the writhing test, respectively. The animals were housed in a controlled environment with constant temperature and humidity and were acclimatized for one week before the experiments were performed (temperature 20–24 °C, humidity 40–45%, 12 h dark/light cycle). All animals were allowed to consume food and water ad libitum. All animal experiments were conducted in accordance with the Gachon University Animal Care and Use Policy (GU1-2022-IA0071-01).

### 4.3. Preparation of RJ Extract

In total, 10 g of RJ root was ground to a powder, mixed to 100 mL of 30% ethanol, and reflux extracted at 85 °C for 3 h. Following three-hour extraction periods, the extracted solution was transferred onto filter paper, filtered, concentrated under low pressure, and then dried in a freeze-dryer. The powdered RJ extract was dissolved in distilled water (DW) for use in all experiments, and the sample yield was 18.32% (e1012).

### 4.4. High-Performance Liquid Chromatography (HPLC)

Polydatin, resveratrol, and emodin were analyzed as RJ components using an Agilent 1100 series HPLC (Agilent, Santa Clara, CA, USA). Before usage, 10 mg of RJ extract was diluted in 1 mL of 50% methanol and filtered through a 0.45 μm syringe filter (Falcon, USA). Polydatin, resveratrol, and emodin were separated by the conditions shown in Table 1.

### 4.5. Preparation of Monosodium iodoAcetate (MIA) Solution and Diet

The purpose of this project was to use MIA to establish an OA model.

Five groups (n = 9 each, total n = 45) of rats were used for the classification: sham, MIA as the negative control, positive control (indomethacin 3 mg/kg; INDO 3, Sigma, USA), and RJ extract (RJ 100 mg/kg and RJ 300 mg/kg). A 40 mg/mL MIA solution was injected 50 ul per rat into the right knee joint cavity to induce OA, establishing an animal model of the condition. Table 2 presents the designs for the OA models during 24 days in sham, MIA, INDO 3, RJ 100, and RJ 300. For this experiment, rats were grouped according to average weight, and the experimenter and evaluator were blinded to the experimental group during data collection.

### 4.6. Weight Bearing (WB) on the Hind Limb

An incapacitance meter (IITC LifeScience Inc., Woodland Hills, CA, USA) was used to measure the OA-induced right hind limb in SD rats from 0 to 24 days after induction. Each animal was measured at least three times to minimize error in the experiment. The following formula was used to calculate the average weight balance for each limb:

WB ratio (%) = (weight on right hind limb/weight on left and right hind limbs) × 100.

### 4.7. Gait Analysis

The OA-induced rats underwent gait analysis behavioral tests on days 7, 14, and 21 of the experiment. To assess gait changes, ink was applied to the ventral surface of both hind limbs, and the rats were allowed to walk on paper to produce at least four clear pairs of footprints. Gait was then evaluated by comparing the footprints of the injured leg to those of the uninjured limb. Paw area and stride length were measured as indicators of pain. For the gait analyses of OA-induced rats, paw area and stride length were measured using ImageJ 1.54d, and all analyses were performed in triplicate.

### 4.8. Cartilage Degradation

After 24 days, OA rats were euthanized using CO_2_. The right knee joint was photographed with a α6600 Sony camera (Sony Corp., Tokyo, Japan). The degree of degradation of the arthritic bone was assessed using macroscopic scoring (Table 3).

### 4.9. Cytokines Analysis in Serum of OA Rats

After 24 days of observation, OA rats were sacrificed and whole blood was obtained for serum analysis. Whole blood was centrifuged to a separate serum and analyzed for the inflammatory cytokines as interleukin (IL)-1β, IL-6, and tumor necrosis factor (TNF)-α. Cytokine assays were analyzed using the MultiAnalyte Kit (R&D Systems Inc., Minneapolis, MN, USA) and Luminex instrument (Luminex Co., Austin, TX, USA). These assays were performed according to the manufacturer’s manual. All experiments were performed in triplicate.

### 4.10. Acetic Acid Induced Writhing Responses (AIW) Pain Model

To establish the AIW model, ICRs were divided into four groups, with eight mice in each group: control, positive control (ibuprofen 200 mg/kg; IBU 200, Sigma, St. Louis, MO, USA), and sample-treated groups (RJ 200 mg/kg and RJ 600 mg/kg) (Table 4). Each sample was provided orally to the mice first, followed by acetic acid intraperitoneally 30 min later. The mice were then given 10 min to induce pain and the pain response is recorded for 10 min. The pain response is quantified by counting the number of writhes on the torso. For this experiment, mice were grouped according to average weight, and the experimenter and evaluator were blinded to the experimental group during data collection.

### 4.11. Cell Culture

The RAW264.7 cell line used for the experiment was bought from the American Type Culture Collection (ATCC, Manassas, VA, USA), and cells were maintained in DMEM media with 10% FBS and 5% penicillin/streptomycin (Gibco, Billings, MT, USA).

### 4.12. Cellviability and Nitric Oxide (NO) Measurement

RAW264.7 cells were seeded in 96 wells the day before the samples were treated and used for the experiments the next day. For cytotoxicity and NO production, dexamethasone 1 µg/mL (DEX 1; Sigma, USA) was used as a positive control, and the experiments were performed at RJ 10, 30, 100, and 300 µg/mL. To check the cytotoxicity, the groups were treated and checked for toxicity with EzCytox (DoGenBio, Seoul, Republic of Korea). The experiments were performed according to the manufacturer’s manual. To determine the amount of NO production, each group of samples was treated with lipopolysaccharide 500 ng/mL (LPS, Sigma, USA) to determine the amount of NO production. The Nitric Oxide Assay Kit (Sigma, USA) was used to determine NO production and was performed according to the manufacturer’s protocol. All experiments were performed in triplicate.

### 4.13. Analisis of Quantitative Real-Time Polymerase Chain Reaction

RNA was isolated from right leg knee cartilage tissue collected from OA rats administrated for 24 days and LPS-induced RAW264.7 cells to determine the expression levels of COX-2, IL-1β, IL-6, NOS2, TNF-α, and MMP13. RNA was analyzed using an RNA Prep Kit (Bioneer, Daejeon, Republic of Korea) and cDNA Convert Mix (Bioneer, Republic of Korea), and experiments were performed according to the manufacturer’s protocol. All experiments were performed in triplicate. The primer sequences prepared for RNA analysis are shown in Table 5 and Table 6.

### 4.14. Protein Analysis Using Western Blot

Protein was isolated from right limb knee cartilage tissue collected from OA rats and LPS-treated RAW264.7 cells to determine the expression levels of inflammatory cytokines. The harvested tissues and cells were lysed in a solution containing RIPA buffer and protease inhibitor (CST Inc., Danvers, MA, USA) and ground with a homogenizer to obtain proteins. The extracted proteins were quantified using a BCA assay (Thermo Fisher Scientific Ltd., Waltham, MA, USA) and loaded on SDS-PAGE. The primary and secondary antibodies used in the experiments were COX-2, IL-1β, IL-6, TNF-α, MMP13, and GAPDH, which were purchased from Abcam, cell signaling technology. All experiments were performed in triplicate.

### 4.15. Statistical Analysis

Statistical analyses were performed using GraphPadPrism ver 9.0 (GraphPad Software, San Diego, CA, USA). Data are presented as mean standard error of the mean, and significance is expressed as *p* value. Statistical analysis was performed by One-way ANOVA followed by Dunnett’s post hoc test and Unpaired *T*-test by Two-tailed *p* value. A Two-way ANOVA, followed by Tukey’s multiple comparisons test, was conducted to evaluate the effects of different doses and treatment groups across various time intervals.

## 5. Conclusions

RJ showed statistically significant improvement in the features of MIA-induced OA in a rat model, including pain, gait dysfunction, and cartilage destruction. Additionally, pro-inflammatory cytokines including IL-1β, IL-6, TNF-α, NOS2, and MMP13 are efficiently downregulated by RJ, thereby assisting in reducing cartilage degradation and relieving pain. These results suggest RJ’s potential as a DMOAD candidate to inhibit chronic OA symptoms and progression, based on an age-related systemic low-grade inflammatory pathology model, warranting further investigation. However, this study is limited by its screening nature; extensive follow-up studies addressing appropriate dosing, safety, specific mechanisms, and more comprehensive OA pathology are required to conclusively address the study objectives. The primary challenges for subsequent studies include determining the optimal dosage of RJ, evaluating its safety in long-term use, and identifying the specific signaling pathways that contribute to its anti-OA effects.

## Figures and Tables

**Table 1:** HPLC analysis condition.

	Condition
Colum	Agilent Zorbax Extend C18 column (250 mm × 4.6 mm, 5 μm; Agilent, Santa Clara, CA, USA)
Mobile phase	(A) 0.1% Phosphoric acid, (B) ACN 0–7 min, 8–20%; 7–12 min, 20–35%; 12–16 min, 35–50%; 16–20 min, 50–65%; 20–24 min, 65–95%; 24–30 min, 95–95%; 30–40 min, 95–8%; 40–45 min, 8–8% (B)
Flow rate	1.0 mL/min
Injection volume	10 μL
Detection wavelength	280 nm
Temperature	35 °C

**Table 2:** OA model design using MIA.

No.	Group Name	OA Model (Intra-Articular; mg/mL, 50 μL)	Diet (AIN-93G)	Final Concentration (mg/kg)
1	sham	Saline	-	-
2	MIA	MIA 40	-	-
3	INDO 3	MIA 40	+indomethacin 0.003%	3
4	RJ 100	MIA 40	+RJ 0.11%	100
5	RJ 300	MIA 40	+RJ 0.33%	300

**Table 3:** Macroscopic score of damaged cartilage.

Grade	Cartilage Appearance
0	Typical appearance of the cartilage surface
1	Slight yellow discoloration or mild fibrillation
2	Erosion in the middle or superficial layer of cartilage is visible
3	Severe deterioration reaching the subchondral bone
4	Massive damage and extensive disclosure of subchondral bone

**Table 4:** AIW model design.

No.	Group Name	AIW Model (Intraperitoneal Injection; 10 mL/kg)	Treatment
1	CT	0.7% acetic acid	DW
2	IBU 200	0.7% acetic acid	ibuprofen 200 mg/kg
3	RJ 200	0.7% acetic acid	RJ 200 mg/kg
4	RJ 600	0.7% acetic acid	RJ 600 mg/kg

**Table 5:** OA rat cartilage tissue primer sequences.

COX-2	F	GTTCCA ACCCAT GTCAAA AC
	R	TGTCAG GAATCT CGGCGT AG
IL-1β	F	AACTCA ACTGTG AAATAG CAGC
	R	TCCACA GCCACA ATGAGT G
IL-6	F	TCCGCA AGAGAC TTCCAG C
	R	CCTCCG ACTTGT GAAGTG G
NOS2	F	AGTCAA CTACAA GCCCCA CG
	R	GCAGCT TGTCCA GGGATT CT
TNF-α	F	GCATGA TCCGAG ATGTGG AA
	R	GATGAG AGGGAG CCCATT TG
MMP13	F	ACCTTC TTCTTG TTGAGT TGGA
	R	CTGCAT TTCTCG GAGTCT A
GAPDH	F	CTTGTG ACAAAG TGGACA TTGTT
	R	TGACCA GCTTCC CATTCT C

COX: cyclooxygenase; IL: interleukin; MMP: matrix metalloproteinase; NOS: nitric oxide synthase; TNF: tumor necrosis factor, GAPDH: Glyceraldehyde 3-phosphate dehydrogenase.

**Table 6:** LPS-treated RAW264.7 cells primer sequins.

COX-2	F	ATCCAT GTCAAA ACCGTG GG
	R	TTGGGG TGGGCT TCAGCA G
IL-1β	F	CCAGCT TCAAAT CTCGCA GC
	R	GTGCTC ATGTCC TCATCC TGG
IL-6	F	CACTTC ACAAGT CGGAGG CT
	R	CAAGTG CATCAT CGTTGT TC
NOS2	F	ACCAAG ATGGCC TGGAGG AA
	R	CCGACC TGATGT TGCCAT TG
TNF-α	F	GAGAAG TTCCCA AATGGC CT
	R	AGCCAC TCCAGC TGCTCC T
MMP8	F	CAATCA ATTCCG GTCTTC GA
	R	GGTTAG CAAGAA ATCACC AGA
MMP13	F	AACCAA GATGTG GAGTGC CT
	R	GACCAG ACCTTG AAGGCT TT
GAPDH	F	ATGGTG AAGGTC GGTGTG
	R	GCCGTG AGTGGA GTCATA C

COX: cyclooxygenase; IL: interleukin; MMP: matrix metalloproteinase; NOS: nitric oxide synthase; TNF: tumor necrosis factor; GAPDH: Glyceraldehyde 3-phosphate dehydrogenase.

## Data Availability

All data from this study are included in the main body of the article.
